# Essential role of miRNAs in orchestrating the biology of the tumor microenvironment

**DOI:** 10.1186/s12943-016-0525-3

**Published:** 2016-05-26

**Authors:** Jamie N. Frediani, Muller Fabbri

**Affiliations:** Children’s Center for Cancer and Blood Diseases and The Saban Research Institute, Children’s Hospital, Los Angeles, Los Angeles, CA USA; Departments of Pediatrics and Molecular Microbiology & Immunology, Norris Comprehensive Cancer Center, Keck School of Medicine, University of Southern California, Los Angeles, CA USA; 4650 Sunset Blvd MS #57, Los Angeles, CA 90027 USA

**Keywords:** Exosomes, microRNAs, Tumor microenvironment, Cancer

## Abstract

MicroRNAs (miRNAs) are emerging as central players in shaping the biology of the Tumor Microenvironment (TME). They do so both by modulating their expression levels within the different cells of the TME and by being shuttled among different cell populations within exosomes and other extracellular vesicles. This review focuses on the state-of-the-art knowledge of the role of miRNAs in the complexity of the TME and highlights limitations and challenges in the field. A better understanding of the mechanisms of action of these fascinating micro molecules will lead to the development of new therapeutic weapons and most importantly, to an improvement in the clinical outcome of cancer patients.

## Background

While cancer treatment and survival have improved worldwide, the need for further understanding of the underlying tumor biology remains. In recent years, there has been a significant shift in scientific focus towards the role of the tumor microenvironment (TME) on the development, growth, and metastatic spread of malignancies. The TME is defined as the surrounding cellular environment enmeshed around the tumor cells including endothelial cells, lymphocytes, macrophages, NK cells, other cells of the immune system, fibroblasts, mesenchymal stem cells (MSCs), and the extracellular matrix (ECM). Each of these components interacts with and influences the tumor cells, continually shifting the balance between pro- and anti-tumor phenotype. One of the predominant methods of communication between these cells is through extracellular vesicles and their microRNA (miRNA) cargo. Extracellular vesicles (EVs) are between 30 nm to a few microns in diameter, are surrounded by a phospholipid bilayer membrane, and are released from a variety of cell types into the local environment. There are three well characterized groups of EVs: 1) exosomes, typically 30–100 nm, 2) microvesicles (or ectosomes), typically 100–1000 nm, and 3) large oncosomes, typically 1–10 μm. Each of these categories has a distinctly unique biogenesis and purpose in cell-cell communication despite the fact that current laboratory methods do not always allow precise differentiation. EVs are found to be enriched with membrane-bound proteins, lipid raft-associated and cytosolic proteins, lipids, DNA, mRNAs, and miRNAs, all of which can be transferred to the recipient cell upon fusion to allow cell-cell communications [1]. Of these, miRNAs have been of particular interest in cancer research, both as modifiers of transcription and translation as well as direct inhibitors or enhancers of key regulatory proteins. These miRNAs are a large family of small non-coding RNAs (19–24 nucleotides) and are known to be aberrantly expressed, both in terms of content as well as number, in both the tumor cells and the cells of the TME. Synthesis of these mature miRNA is a complex process, starting with the transcription of long, capped, and polyadenylated pri-miRNA by RNA polymerase II. These are cropped into a 60–100 nucleotide hairpin-structure pre-miRNA by the microprocessor, a heterodimer of Drosha (a ribonuclease III enzyme) and DGCR8 (DiGeorge syndrome critical region gene 8). The pre-miRNA is then exported to the cytoplasm by exportin 5, cleaved by Dicer, and separated into single strands by helicases. The now mature miRNA are incorporated into the RNA-induced silencing complex (RISC), a cytoplasmic effector machine of the miRNA pathway. The primary mechanism of action of the mature miRNA-RISC complex is through their binding to the 3’ untranslated region, or less commonly the 5’ untranslated region, of target mRNA, leading to protein downregulation either via translational repression or mRNA degradation. More recently, it has been shown that miRNAs can also up-regulate the expression of target genes [2]. MiRNA genes are mostly inter-genic and are transcribed by independent promoters [3] but can also be encoded by introns, sharing the same promoter of their host gene [4]. MiRNAs undergo the same regulatory mechanisms of any other protein coding gene (promoter methylation, histone modifications, etc.…) [5, 6]. Interestingly, each miRNA may have contradictory effects both within varying tumor cell lines and within different cells of the TME. In this review, we provide a state-of-the-art description of the key role that miRNAs have in the communication between tumor cells and the TME and their subsequent effects on the malignant phenotype. Finally, this review has made every effort to clarify, whenever possible, whether the reference is to the −3p or the -5p miRNA. Whenever such clarification has not been provided, this indicates that it was not possible to infer such information from the cited bibliography.

## Angiogenesis and miRNAs

Cellular plasticity, critical in the development of malignancy, includes the many diverse mechanisms elicited by cancer cells to increase their malignant potential and develop increasing treatment resistance. One such mechanism, angiogenesis, is critical to the development of metastatic disease, affecting both the growth of malignant cells locally and their survival at distant sites. In the last ten years, miRNAs, often packaged in tumor cell-derived exosomes, have emerged as important contributors to the complicated regulation and balance of pro- and anti-angiogenic factors.

**Fig. 1:**
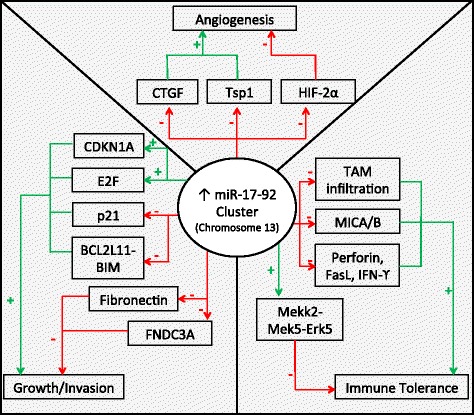
Central role of the miR-17-92 cluster in the biology of the TME. The miR-17-92 cluster encoding miR-17, −18a, −19b, −20a, and -92a is upregulated in multiple tumor types and interacts with various components of the TME to finely “tune” the TME through a complex combination of pro- and anti-tumoral effects

Most commonly, miRNAs derived from cancer cells have oncogenic activity, promoting angiogenesis and tumor growth and survival. The most-well characterized of the pro-angiogenic miRNAs, the miR-17-92 cluster encoding six miRNAs (miR-17, −18a, −19a, −19b, −20a, and −92a), is found on chromosome 13, and is highly conserved among vertebrates [7]. The complex and multifaceted functions of the miR-17-92 cluster are summarized in Fig. 1. Amplification, both at the genetic and RNA level, of miR-17-92 was initially found in several lymphoma cell lines and has subsequently been observed in multiple mouse tumor models [7]. Up-regulation of this particular locus has further been confirmed in miRnome analysis across multiple different tumor types, including lung, breast, stomach, prostate, colon, and pancreatic cancer [8]. The miR-17-92 cluster is directly activated by Myc and modulates a variety of downstream transcription factors important in cell cycle regulation and apoptosis including activation of E2F family and Cyclin-dependent kinase inhibitor (CDKN1A) and downregulation of BCL2L11/BIM and p21 [7]. In addition to promoting cell cycle progression and inhibiting apoptosis, the miR-17-92 cluster also downregulates thrombospondin-1 (Tsp1) and connective tissue growth factor (CTGF), important antiangiogenic proteins [7]. Similarly, microvesicles from colorectal cancer cells contain miR-1246 and TGF-β which are transferred to endothelial cells to silence promyelocytic leukemia protein (PML) and activate Smad 1/5/8 signaling promoting proliferation and migration [9]. Likewise, lung cancer cell line derived microvesicles contain miR-494, in response to hypoxia, which targets PTEN in the endothelial cells promoting angiogenesis through the Akt/eNOS pathway [10]. Lastly, exosomal miR-135b from multiple myeloma cells suppresses the HIF-1/FIH-1 pathway in endothelial cells, increasing angiogenesis [11]. A summary of the studies showing the functions of exosomal miRNAs in shaping the biology of the TME is provided in Table 1.

**Table 1:** Actions of exosomal miRNAs exchanged between cells of the TME

Angiogenesis:					
miRNA	Cell of origin	Accepting cell	Pathway/target	Effect on TME	Ref.
miR-135b	Multiple myeloma	Endothelial cells	HIF-1/FIH-1	↑angiogenesis	[11]
miR-494	Lung cancer	Endothelial cells	PTEN/AKT/eNOS	↑angiogenesis	[10]
miR-503	Endothelial cells	Breast cancer	Cyclin D2 and D3	↓Tumor growth and invasion	[22]
miR-1246	Colorectal cancer	Endothelial Cells	PML/Smad 1/5/8	↑ Growth & migration	[9]
Stromal compartment:					
miR-105	Breast cancer	Endothelial cells	ZO-1	↓Tight junctions ↑Metastatic progression	[68]
miR-202-3p	CLL	Stromal cells	c-fos/ATM	↑Tumor growth	[53]
Immune system:					
miR-29a	NSCLC	TAM	TLR8/NF-κB	↑Growth & metastasis	[75]
miR-21	NSCLC	TAM	TLR8/NF-κB	↑Growth & metastasis	[75]
	NBL	TAM	TLR8/NF-κB	↑miR-155	[76]
miR-155	TAM	NBL	TERF1	↑ Drug resistance	[76]
miR-23a	Hypoxic tumor derived	NK cells	CD107a	↓ NK cell response	[95]
miR-210					
miR-214	Tumor cells (various)	Regulatory T cells	PTEN	↑Immunosuppression	[96]
miR-223	TAM	Breast cancer	Mef2c/β-catenin	↑ Invasion	[82]

*Abbreviations*: *TAMs* Tumor Associated Macrophages, *CLL* chronic lymphocytic leukemia, *NSCLC* non-small cell lung cancer, *NBL* Neuroblastoma

The most common target of anti-angiogenic therapy is VEGF, and not unsurprisingly, multiple miRNAs (including miR-9, miR-20b, miR-130, miR-150, and miR-497) promote angiogenesis through the induction of the VEGF pathway. The most studied of these is the up-regulation of miR-9 which has been linked to a poor prognosis in multiple tumor types, including breast cancer, non-small cell lung cancer, and melanoma [12]. The two oncogenes MYC and MYCN activate miR-9 and cause E-cadherin downregulation resulting in the upregulated transcription of VEGF [13]. In addition, miR-9 has been shown to upregulate the JAK-STAT pathway, supporting endothelial cell migration and tumor angiogenesis [13]. Both amplification of miR-20b and miR-130 as well as miR-497 suppression regulate VEGF through hypoxia inducible factor 1α (HIF-1α) supporting increased angiogenesis [14–17]. Similarly, miR-146a suppression as often seen in hepatocellular carcinoma (HCC), leads to increased VEGF expression through downregulation of APC and upregulation of HAb18G, thereby portending a poor prognosis [18]. Two further miRNAs promoting angiogenesis are similarly induced by the hypoxic TME. The first, miR-210 is an important mediator of the endothelial cell response to low oxygen tension, down-regulating normoxic genes and activating MYC to stimulate cell cycle progression [19]. Studies on various primary tumors types have shown that elevated circulating miR-210 levels are both a potential diagnostic tool as well as a poor prognostic indicator at diagnosis [19, 20]. Similarly, hypoxia induces miR-424 expression in endothelial cells, which targets cullin 2 (CUL2), a scaffolding protein essential to the ubiquitin ligase system, thus increasing HIF-α levels [21]. While much of the literature has focused on the effect of cancer cells on the endothelium, there is also emerging evidence that miRNAs can be transferred to the cancer cells from the endothelium. One such miRNA, miR-503 is released in endothelial exosomes and upon uptake by the breast cancer cells limits tumor growth and invasion by inhibiting Cyclin D2 and D3 [22].

While many miRNAs provide a pro-angiogenic advantage to the malignant cells, in certain environments, miRNAs have also been found to be tumor suppressors. The most well-known anti-angiogenic miRNA is the miR-200 family which inhibits angiogenesis by targeting interleukin-8 and CXCL1 [23]. With enhanced delivery of miR-200 into the tumor endothelium of multiple cancer models including ovarian, lung, renal and basal-like breast cancers, a significant reduction in metastasis formation and angiogenesis was noticed [23]. Similarly, miR-98 expression downregulates activin receptor-like kinase-4 (ALK4) and metalloproteinase-11 (MMP11) expression, and miR-506 downregulates Sphingosine kinase 1 (SPHK1), thereby inhibiting cell growth and angiogenesis in breast cancer and hepatoma cell models, respectively [24, 25]. Additionally, miR-126 regulates endothelial cell recruitment and vessel formation through inhibition of Insulin-like growth factor binding protein 2 (IGFBP2) and c-Mer tyrosine kinase (MERTK) signaling [26]. Low expression of miR-126 has been correlated to increased microvessel density and worse disease prognosis in multiple cancer types including non-small cell lung cancer, glioblastoma, breast cancer, and gastric cancer [27]. Lastly, miR-542-3p has been found to inhibit translation of Angiopoietin-2 mRNA, a potent hypoxia-induced regulator of endothelial cell proliferation and migration [28]. Elevated levels of miR-542-3p cause attenuated endothelial cell angiogenesis in culture and reduced tumor burden in mouse models [28]. Clinical prognosis in patients with Stage III/IV breast carcinoma is inversely correlated with miR-542-3p levels [28].

## Stroma and miRNAs

Angiogenesis is essential to the development of tumor growth and metastatic disease, however, without the stroma, the tumor cells would be unable to establish a favorable niche. Fibroblasts, the predominant cellular feature of stroma, are responsible for the formation and maintenance of the extracellular matrix. Additionally, stromal cells, including fibroblasts, perivascular cells, and mesenchymal stem cells, generate growth factors to assist in tumor growth and progression, provide a source of matrix-remodeling proteins, and influence tumor angiogenesis [29]. Recent discoveries have found that tumor-derived exosomes modify the cellular phenotype of fibroblasts to Cancer-Associated Fibroblasts (CAF). CAFs have higher expression of α-smooth muscle actin and secrete a combination of increased growth factors to promote local epithelial cell growth, increased extracellular matrix (ECM) degrading proteases facilitating ECM turnover and changes in composition, and increased modulation of the immune response after tissue injury [30]. This change in phenotype promotes the growth, invasion, and metastatic potential of the tumor cells and is a critical step in the progression of malignancy. In addition to transitioning the fibroblasts to CAFs, miRNAs can trigger the molecular process leading epithelial cells to become phenotypically mesenchymal cells; this phenomenon is known as the epithelial-mesenchymal transition (EMT) [31].

The pivotal discovery in 2012 by Mitra et al. laid the ground-work for our current knowledge on the interactions between tumor-derived miRNAs and fibroblasts. In combination, the down-regulation of miR-214 and miR-31 and the up-regulation of miR-155 trigger the reprogramming of quiescent fibroblasts to CAFs [32]. As expected, the reverse regulation of these miRNAs reduced the migration and invasion of co-cultured ovarian cancer cells [32]. While the pathway of miR-155’s involvement in CAF biology is still being elucidated, the pathways of miR-214 and miR-31 have been established. In endometrial cancer, miR-31 was found to target the homeobox gene SATB2, leading to enhanced tumor cell migration and invasion [33]. MiR-214 similarly has an inverse correlation with its chemokine target, C-C motif Ligand 5 (CCL5) [32]. CCL5 secretion has been associated with enhanced motility, invasion, and metastatic potential through NF-κB-mediated MMP9 activation and through generation and differentiation of myeloid-derived suppressor cells (MDSCs) [34–36]. Furthermore, miR-210 and miR-133b overexpression and miR-149 suppression have been subsequently found to independently trigger the conversion to CAFs, possibly through paracrine stimulation, and to additionally promote EMT in prostate and gastric cancer, respectively [37–39]. MiR-210 additionally enlists monocytes and encourages angiogenesis [37]. Similarly, miR-409-3p/-5p overexpression has also been shown in prostate cancer to induce the EMT, promote cell growth, and repress tumor suppressors, notably RSU1 and STAG2 [40]. Lastly, in addition to promotion of angiogenesis as noted above, the miR-200 family is overexpressed in many solid tumor cell lines, particularly breast cancer, and is secreted into extracellular vesicles to act as an important regulator of the EMT [41].

Once the fibroblasts have been switched to the CAF phenotype, they serve two important roles in the TME. First, they secrete increased growth factors, including hepatocyte growth factor (HGF), insulin-like growth factor (IGF), nerve growth factor (NGF), WNT1, EGF, FGF2, VEGF, and PDGF [42]. These growth factors promote cancer cell growth and are critical to the cross-talk between epithelial cells and fibroblasts. One of the first miRNA clusters studied in CAFs was the miR-15a/16-1 cluster which is notably down-regulated in CAFs in patient samples of prostate cancer [43]. Decreased levels of these two miRNAs led to decreased post-transcriptional repression of FGF-2 and FGFR-1 causing enhanced cancer cell survival [43]. Further work has shown that miR-15a and miR-16-1 act as tumor suppressors and when decreased lead to increased levels of BCL2, CCNDI, and WNT3A in prostate cancer, increased BCL2 in Chronic Lymphocytic Leukemia, and increased IGSF4 in leukemia [44–46]. Similarly, miR-148a has been shown to be down-regulated in endometrial cancer CAFs with direct upregulation of WNT10B leading to increased cancer cell motility [47]. Previous work elucidating miRNA transcriptomes of various tumor types had shown down-regulation of miR-148a to be part of the miRNA signature of tumors with increased metastatic potential [48]. Additionally, miR-21 up-regulation occurs predominantly in CAFs in breast, lung, colon, and esophageal cancers and has been shown to contribute to inducing the CAF phenotype and increase the invasiveness and migration of the associated tumor cells [49]. Similarly, miR-101 is notably down-regulated in CAFs causing increased expression of CXCL12 affecting migration and invasiveness of lung cancer cells [50]. Additionally, repression of PTEN expression in CAFs leads to down-regulation of miR-320 and up-regulation of its oncogene target ETS2, inducing a pro-oncogenic environment [51]. While low miR-126 expression has been shown to cause increased angiogenesis as noted previously, low miR-126 expression has also been shown to correlate with poor metastasis-free survival of breast cancer patients due to stromal cell recruitment [52]. Suppressed miR-126/126* levels lead to an increased levels of both SDF-1α and CCL2 thereby promoting tumor cell proliferation and recruitment of MSCs and inflammatory monocytes in the TME [52]. Lastly, Chronic Lymphoblastic Leukemia (CLL)-derived exosomes are enriched with miR-202-3p, which when transferred to the stromal cells, increases c-fos and ATM levels as well as affects cellular Sufu levels leading to increased tumor proliferation [53].

Another function of CAFs is the destruction of the ECM and its remodeling with a tumor-supportive composition and structure which includes modulation of specific integrins and metalloproteinases as some of the most studied miRNA targets. The 23 matrix metalloproteinases (MMPs) are critical in the ECM degradation, disruption of the growth signal balance, resistance to apoptosis, establishment of a favorable metastatic niche, and promotion of angiogenesis [54]. As expected, miRNAs have been found to regulate the actions of MMPs, together working to promote cancer cell growth, invasiveness, and metastasis. In HCC, MMP2 and 9 expression is up-regulated by miR-21 via PTEN pathway downregulation. Similarly, in cholangiocarcinoma it was observed that reduced levels of miR-138 induced up-regulation of RhoC, leading to increased levels of the same two MMPs [55, 56]. Conversely, miR-29b suppresses MMP2 and 9, thus loss of miR-29b expression as seen in breast cancer, causes increased MMP2 and 9 [57]. Similarly, placental growth factor (PLGF) suppresses miR-543 and miR-543 which inhibits MMP7 translation, therefore high levels of PLGF as seen in ovarian cancer specimens increase MMP7 levels and thus the cancer’s invasion ability [58]. Likewise, MMP2 and MMP11, in addition to pErk and ADAM15, are upregulated due to miR-24’s modulation of the EGFR pathway [59]. Working in concert, elevated levels of miR-21 additionally have been shown to cause downregulation of MMP inhibitors RECK and TIMP3 in gliomas and elevated miR-24 down-regulates TIMP2 in breast cancer models, increasing the tumor cells migratory and invasive capabilities [59, 60]. In addition to MMPs, integrins, heterodimeric receptors responsible for anchoring cells to ECM proteins and for signal transduction, are similarly vital to the TME. Several miRNAs have been found in multiple tumor models to target integrins including miR-93 suppression of integrin-β8, miR-183, miR-124, and miR-29b suppression of integrin-β1, and miR-29b suppression of integrin-α6 [57, 61–63]. Lastly, miRNAs have also been found to directly affect the expression of stromal components. The presence of miR-17 represses the expression of both fibronectin and fibronectin type-III domain containing 3A (FNDC3A) which decreases cell adhesion and migration [64]. In HCC, miR let-7 g is inversely correlated with Type 1 collagen α2 levels and low miR let-7 g levels are directly correlated with poor survival [65]. Similarly, decreased let-7d in Renal Cell Carcinoma is associated with advanced tumor stage and increased vascular invasion secondary to Collagen Type III α1 and CCL7 activation [66]. In addition, miR-26b, commonly down-regulated in CAFs, enhances Type XII Collagen α1 production stimulating epithelial invasion in breast cancer [67]. Lastly, metastatic breast cancer cells secrete miR-105 in exosomes which targets tight junction protein ZO-1 in endothelial cells promoting metastatic progression [68].

## Immune system activation and miRNAs

In addition to the hallmarks of promoting angiogenesis and a favorable stromal environment for growth and metastasis, miRNAs also play a critical role in shaping an inflammatory TME. Infiltrating immune system cells can operate both as pro- and anti-tumoral components of the TME [69]. In recent years, the presence of increased inflammatory cell infiltration, particularly Tumor Associated Macrophages (TAMs), has been linked to worse disease prognosis in most tumor types [70, 71].

TAMs are macrophages, derived predominantly from bone marrow monocytes, which are recruited by TME-derived CCL2 to infiltrate tumor tissues [72]. Within the TME, TAMs are educated by the surrounding tumor cells and can either adopt the M1-polarized phenotype which produces pro-inflammatory, antitumor cytokines (including IL-12 and IL-23), or the M2-polarized phenotype which is immunosuppressive and critical in tissue repair [73]. Particularly in advanced cancers, TAMs are more frequently educated to the M2-polarized phenotype, creating an immunosuppressive environment and thereby stimulating tumor progression and metastasis [73]. However, heterogeneity of TAMs within the TME has been increasingly demonstrated with the presence of M1-polarized macrophages found in many tumors, particularly early stage and regressing tumors [74]. The first complete demonstration of the cross-talk communication of miRNAs between the TAMs and tumor cells was shown in lung cancer. Non-small cell lung cancer cells were shown to secrete miR-21 and miR-29a in exosomes. These two miRNAs are then taken up by surrounding TAMs in the TME and bind to Toll-like receptor 8 (TLR8) located inside the TAM’s endosomes. This work provides the first demonstration of the existence of miRceptors, defined as proteic receptors for miRNAs, whose signaling is triggered by their binding to specific miRNAs, in a ligand-receptor fashion [75]. As a consequence of exosomic miR-21 and miR-29a binding to TLR8, TAMs secrete increased IL-6 and TNF-α, creating a sterile inflammatory TME that promotes cancer growth and dissemination. More recently, the implications of this mechanism were extended to Neuroblastoma and other malignancies, where it was shown that cancer cell derived exosomic miR-21, by binding to TLR8 in surrounding TAMs, up-regulated the levels of miR-155 in TAMs and TAM-derived exosomes which were then transferred back to the cancer cells  where the telomerase inhibitor TERF1 was silenced. Silencing of TERF1 led to increased Cisplatin resistance in both in vitro and in vivo xenografts [8, 76–80]. Increased levels of miR-21 in body fluids and biopsies has been extensively reported as a prognostic and predictive-of-response-to-treatment marker for several types of cancers [81]. A similar mechanism showed M2 TAM-derived exosomes containing miR-223 are delivered to breast cancer cells and promote invasiveness [82]. The breast cancer cell invasiveness was successfully reversed when antisense miR-223 was transfected into the tumor cells [82]. Additionally, TAMs secrete CCL18 which reduces miR-98 and miR-27b expression via the Ras/ERK/PI3K/AKT/NFκB/Lin28b signaling pathway in tumor cells, thereby enhancing the epithelial-mesenchymal transition and metastasis of breast cancer cells [83]. Furthermore, miRNAs have been shown to directly affect the number of infiltrating TAMs in the case of miR-92a suppression in breast cancer and the protection against apoptosis of tumor-promoting M2-phenotype TAMs as evidence by miR-142-3p downregulation in glioblastoma, both correlating with overall survival [84, 85]. Similarly, IL-6 released from the tumor cells induce miR-17 and miR-20a down-regulation in TAMs which induces HIF-2α and transcription of proangiogenic genes [86]. Lastly, TAMs with suppression of miR-146a and miR-222 demonstrate promotion of M2-type polarization and macrophage chemotaxis in breast cancer models, respectively [87]. While some TAM-derived miRNAs increase the growth and invasive potential of the tumor cells, miR-125b has been show to promote macrophage activation, exemplified by increased T cell activation, increased sensitivity to IFN-γ, and more effective tumor cell killing [88]. Down-regulation of miR-125b has been described in many solid tumors, including melanoma, squamous cell carcinomas of the mouth and tongue, as well as ovarian, breast, and prostate cancers [88]. Similarly, miR-23a/27a/24-2 cluster regulates M1- and M2- polarization through a negative feedback loop [89]. This miRNA cluster has been shown to be downregulated in the TAMs of breast cancer patients, thereby promoting tumor cell growth [89]. Alternatively, miR-511-3p triggers a negative-feedback response to upregulation of MRC1 during differentiation of pro-tumoral TAMs, mitigating the TAMs pro-tumoral potential [90].

While TAMs are at the moment the most widely-studied inflammatory components of the TME, miRNAs also play a role in the biology of NK cells and T lymphocytes. Predominantly, miRNAs function to shift the local inflammatory TME to be immunosuppressive and immune tolerant, allowing cancer cells to grow and metastasize, escaping immune surveillance. Tumor-derived TGF-β induces miR-183 in NK cells thereby suppressing DAP12 transcription, a necessary protein in NK-mediated tumor cell death [91]. Consistent with these findings, lower levels of DAP12 were observed in the tumor-infiltrating NK cells of multiple lung cancer subtypes. Working in concert, miR-146a overexpression secondary to constitutive STAT3 expression in HCC, suppresses anti-tumor response by NK cells and cytotoxic T lymphocytes through increased inflammatory cytokines and TGF-β, leading to increased cell growth [92]. Similarly, miR-92a is released from glioma cells and induces NK cell expression of IL-6 and IL-10, significantly attenuating the expression of NK cell-derived anti-tumor molecules, including perforin, Fas ligand, and IFN-γ [93]. Furthermore, miR-210 has also been shown to silence PTPN1, HOXA1, and TP53I11 transcription, decreasing the tumor’s susceptibility to Cytotoxic T-lymphocyte killing [94]. Moreover, miR-23a in addition to miR-210 and TGF-β, occur in high concentrations in hypoxic tumor-derived microvesicles with miR-23a directly targeting CD107a expression in NK cells, decreasing the NK cell response [95]. Lastly, increased secretion of miR-214 in microvesicles from various human cancers leads to downregulated PTEN in the regulatory T cells promoting Treg expansion and enhanced immunosuppression in the TME [96]. While miRNA can alter NK cell and T lymphocyte function and their ability to kill, miRNAs have also been shown to change the cell surface ligands on tumor cells to allow for immune evasion from the NK cell receptor NKG2D. In both human breast cancer stem cells and ovarian cancer, overexpression of miR-20a has been shown to promote immune evasion from NK cells through decreased transcription of MICA/B proteins, the ligand of the NKG2D receptor on NK and cytotoxic T-cells [97, 98]. Similarly miR-10b directly binds MICB and downregulates its membrane expression, decreasing NKG2D-mediated tumor killing [99]. Contradicting these findings, miR-17/20a overexpression has also been shown to be a tumor suppressor by enhancing NK-cell detection via Mekk2-Mek5-Erk5 pathway in breast and colon primary tumors [100]. Moreover, miRNAs can change the expression of NKG2D immunoreceptor ligands inhibiting NK and T cell tumor killing as seen in miR-29 downregulation in many solid tumor lines causing upregulation of B7-H3 expression and miR-34a/c upregulation decreasing ULBP2 expression [101, 102]. Further studies are warranted to clarify the role of the miR-17-92 cluster and other miRNAs in the NK-mediated immunologic response within the TME.

## Conclusions and future directions

As has been shown throughout this review, miRNAs have an important and varied effect on human carcinogenesis by shaping the biology of the TME towards a more permissive pro-tumoral phenotype. The complex events leading to such an outcome are currently quite universally defined as the “educational” process of cancer cells on the surrounding TME. While the initial focus was on the direction from the cancer cell to the surrounding TME, increasingly interest is centered on the implications of a more dynamic bidirectional exchange of genetic information. MiRNAs represent only part of the cargo of the extracellular vesicles, but an increasing scientific literature points towards their pivotal role in creating the micro-environmental conditions for cancer cell growth and dissemination. The nearby future will have to address several questions still unanswered. First, it is absolutely necessary to clarify which miRNAs and to what extent they are involved in this process. The contradictory results of some studies can be explained by the differences in tumor-types and by different concentrations of miRNAs used for functional studies. Understanding whether different concentrations of the same miRNA elicit different target effects and therefore changes the biology of the TME, will represent a significant consideration in the development of this field. It is certainly very attractive (especially in an attempt to develop new and desperately needed better cancer biomarkers) to think that concentrations of miRNAs within the TME are reflected systemically in the circulating levels of that same miRNA, however this has not yet been irrefutably demonstrated. Moreover, the study of the paracrine interactions among different cell populations of the TME and their reciprocal effects has been limited to two, maximum three cell populations. This is still way too far from describing the complexity of the TME and only the development of new tridimensional models of the TME will be able to cast a more conclusive light on such complexity. Finally, the pharmacokinetics of miRNA-containing vesicles is in its infancy at best, and needs to be further developed if the goal is development of new therapies based on the use of exosomic miRNAs. Therefore, the future of miRNA research, particularly in its role in the TME, holds still a lot of questions that need answering. However, for these exact same reasons, this is an incredibly exciting time for research in this field. We can envision a not too far future in which these concerns will be satisfactorily addressed and our understanding of the role of miRNAs within the TME will allow us to use them as new therapeutic weapons to successfully improve the clinical outcome of cancer patients.
